# Investigating antimicrobial resistance genes in probiotic products for companion animals

**DOI:** 10.3389/fvets.2024.1464351

**Published:** 2024-10-22

**Authors:** Adam Kerek, Emese Szabó, Ábel Szabó, Márton Papp, Krisztián Bányai, Gábor Kardos, Eszter Kaszab, Krisztina Bali, Ákos Jerzsele

**Affiliations:** ^1^Department of Pharmacology and Toxicology, University of Veterinary Medicine, Budapest, Hungary; ^2^National Laboratory of Infectious Animal Diseases, Antimicrobial Resistance, Veterinary Public Health and Food Chain Safety, University of Veterinary Medicine, Budapest, Hungary; ^3^Centre for Bioinformatics, University of Veterinary Medicine, Budapest, Hungary; ^4^Veterinary Medical Research Institute, Budapest, Hungary; ^5^One Health Institute, Faculty of Health Sciences, University of Debrecen, Debrecen, Hungary; ^6^National Public Health Center, Budapest, Hungary; ^7^Department of Metagenomics, University of Debrecen, Debrecen, Hungary; ^8^Department of Microbiology and Infectious Diseases, University of Veterinary Medicine, Budapest, Hungary

**Keywords:** probiotics, ARG, NGS, companion animals, antimicrobial resistance

## Abstract

**Introduction:**

One of the greatest challenges of our time is antimicrobial resistance, which could become the leading cause of death globally within a few decades. In the context of One Health, it is in the common interest to mitigate the global spread of antimicrobial resistance by seeking alternative solutions, alongside appropriate drug selection and responsible use. Probiotics offer a potential avenue to reduce antibiotic usage; however, there is a scarcity of research that examines commercial products in terms of carrying antimicrobial resistance genes (ARGs) involved in resistance development through microbial vectors.

**Methods:**

Our study investigated 10 commercially available probiotic products for cats and dogs. Initially, we conducted phenotypic testing through determination of minimum inhibitory concentration (MIC) for antibiotics important in animal and public health. Subsequently, we performed next-generation sequencing (NGS) of the products to elucidate the genetic background behind the decrease in phenotypic sensitivity.

**Results:**

In total, 19 types of ARGs were identified, with 57.9% being found on plasmids, and in two cases, carriage as mobile genetic elements were found. One of the genes identified was the *APH(3*′*)-Ia* gene, capable of inactivating aminoglycoside antibiotics through phosphotransferase enzyme production regulation, while the other was the *tetS* gene, capable of conferring reduced sensitivity to tetracycline antibiotics through target protection.

**Discussion:**

Our findings underscore the importance of approaching antimicrobial resistance investigations from a broader perspective. We suggest that further studies in this area are justified and raise questions regarding the need to extend legally required studies on probiotic products from their use in economic livestock to their use in companion animals.

## 1 Introduction

Antimicrobial resistance is the property of bacteria enabling them to survive targeted treatment with antibiotics (1). Nowadays, infections caused by resistant and multi-resistant strains are spreading increasingly (2) which, according to the World Health Organization (WHO), will become one of the 10 leading threats to global health in the twenty-first century (3).

Probiotics are living microorganisms that, upon entering the body, exert beneficial effects on the entire host organism. The majority belong to bacterial strains which occur naturally in the gut. Species belonging to the *Enterococcus, Lactobacillus*, and *Bifidobacterium* genera are commonly used for this purpose, which is the maintenance or restoration of gut microbiome balance (4). Although numerous studies regarding their effects on the human body have been conducted, significantly fewer investigations have been undertaken on their effects on companion animals. Besides the intestinal effects, it is likely that probiotics exert immunomodulatory and stress-reducing effects in companion animals and assist in balancing digestion-related blood parameters (5). Certain probiotic strains also exhibit antimicrobial effects against specific pathogenic gastrointestinal bacteria (6). Existing research suggests that the effects of probiotics are most favorable for bacteria species specific to the host, especially in gastrointestinal diseases (7). For probiotic organisms, it may be desirable to have antibacterial resistance to survive in antibiotic treatment. However, bacteria can transfer resistance genes to each other vertically or horizontally, as a result they may be able to transfer resistant genes to pathogenic bacteria. The mammalian gastrointestinal tract provides favorable conditions for gene transfer. In the case of products for livestock, regulations prohibit the inclusion of resistance genes relevant from a public health perspective, but such regulation is lacking for companion animals (8).

During the examination of the *Bacillus amyloliquefaciens* strain used as a probiotic, it was found that although it was sensitive to fluoroquinolones, it exhibited reduced sensitivity to older antibacterial groups (9). However, another study did not detect resistance genes in this species (10). Intrinsic resistance is characteristic of *Lactobacillus* species, which generally show resistance to multiple antibiotic groups, such as aminoglycosides, glycopeptides, nucleic acid synthesis inhibitors, and folate synthesis inhibitors (11). Resistance genes against penicillin have been detected in various isolates in several studies (12–14). Oxacillin and cephalosporin resistance have also been detected in *Lactobacillus plantarum* and *Lactobacillus rhamnosus* (15, 16). Mater et al. demonstrated that *Lactobacillus acidophilus* strains are capable of acquiring vancomycin resistance genes from *Enterococcus* species via horizontal gene transfer (17). The most common resistance genes in *Lactobacillus* species confer resistance to tetracyclines and macrolides. The *tetM* and *tetS* genes can be found on both plasmids and chromosomes. However, the *tetL* gene is only described on plasmids. The *ermB* gene responsible for resistance to erythromycin is also found on plasmids (18). It has also been shown that strains with more than one tetracycline resistance gene exhibit greater resistance, as these genes have a synergistic effect (19). Feld et al. isolated a *Lactobacillus plantarum* strain with a *tetM* gene transposon capable of transferring it to other lactic acid-producing bacteria (20).

In a subsequent study, examining *Pediococcus acidilactici* strains against 21 different antimicrobial agents (penicillin, oxacillin, ampicillin, piperacillin, imipenem, vancomycin, streptomycin, gentamicin, amikacin, kanamycin, tetracycline, chloramphenicol, minocycline, doxycycline, cotrimoxazole, azithromycin, erythromycin, clindamycin, norfloxacin, ciprofloxacin, and levofloxacin), it was found that only four strains exhibited sensitivity to piperacillin, imipenem, chloramphenicol, and erythromycin. While they showed only moderate resistance to clindamycin, doxycycline, and levofloxacin, they were fully resistant to the remaining agents (21). In another investigation, a *Pediococcus pentosaceus* strain tested against 19 agents was only resistant to ceftazidime and sulfamethoxazole. In contrast, an *Enterococcus faecalis* strain was almost entirely resistant to all agents. Based on this, *Pediococcus* species appear to be safer in terms of resistance as probiotics compared to *Enterococcus* species, meaning they are less likely to contribute to the spread of antimicrobial resistance (22). However, *Enterococcus faecium* is the most common component of probiotic preparations for companion animals. A probiotic strain examined by Bs et al. was found to be resistant to penicillin, ampicillin, erythromycin, kanamycin, and streptomycin. However, it was sensitive to tetracycline, chloramphenicol, and rifampicin (23).

Therefore, it is particularly important to adequately regulate probiotic products marketed for companion animals in terms of antimicrobial resistance gene carriage. In our study, we investigate the most common probiotic products for companion animals available in Hungary through next-generation sequencing and compare the results with the phenotypic resistance profiles of strains isolated from these products.

## 2 Materials and methods

### 2.1 The origin of the products and strains

We purchased 10 probiotic products available for dogs and cats from retailers selling veterinary products in Hungary. The isolation of strains indicated on the products was carried out by the Department of Microbiology and Infectious Diseases at the University of Veterinary Medicine Budapest. The isolation of *Enterococcus faecium* strains was successful in all nine of the products listing it (9/9). Among the other strains listed on the products, we were able to isolate *Lactobacillus plantarum* in one case (1/4), *Pediococcus pentasaceus* in two cases (2/2), and *Pediococcus acidilactici* in one case (1/2). Additionally, certain products indicated the presence of *Pediococcus* strains (one strain) and *Lactococcus* strains (eight strains) in deactivated form. The species identity of the strains was determined using MALDI-TOF mass spectrometry (Bruker, Mannheim, Germany). The isolates were used for minimal inhibitory concentration (MIC) testing. The properties of each product are summarized in Table 1.

**Table 1:** The examined products, the strains contained within them along with their strain numbers, and the colony-forming units (CFU) present in the products.

**Product**	**Strain**	**Strain number**	**CFU/g**
A-product	*Lactobacillus fermentum*	NCIMB 41636	3^*^10^11^
	*Lactobacillus plantarum*	NCIMB 41638	
	*Lactobacillus rhamnosus*	NCIMB 41640	
B-product	*Enterococcus faecium*	NCIMB 10415	1.125^*^10^8^
C-product	*Enterococcus faecium*	NCIMB 10415	1^*^10^13^
D-product	*Lactobacillus plantarum*	DSM 12837	1^*^10^9^
	*Pediococcus acidilactici*	DSM 16243	
	*Enterococcus faecium*	NCIMB 10415	
E-product	*Lactobacillus plantarum*	DSM 12837	1^*^10^9^
	*Pediococcus acidilactici*	DSM 16243	
	*Enterococcus faecium*	NCIMB 10415	
F-product	*Pediococcus pentasaceus*	DSM 1283U	1^*^10^9^
	*Lactobacillus brevis*	DSM 12835	
	*Lactobacillus buchnerii*	DSM 12856	
	*Lactobacillus plantarum*	DSM 12836	
	*Lactobacillus rhamnosus*	NCIMB 30121	
	*Enterococcus faecium*	NCIMB 10415	2.8^*^10^11^
	*Lactobacillus acidophilus*	CECTU 529	8.6^*^10^12^
G-product	*Pediococcus pentasaecus*	DSM 1283U	1^*^10^9^
	*Lactobacillus brevis*	DSM 12835	
	*Lactobacillus buchnerii*	DSM 12856	
	*Lactobacillus plantarum*	DSM 12836	
	*Lactobacillus rhamnosus*	NCIMB 30121	
	*Enterococcus faecium*	NCIMB 10415	1.7^*^10^11^
	*Lactobacillus acidophilus*	CECTU 529	5.2^*^10^12^
H-product	*Enterococcus faecium*	NCIMB 10415	2^*^10^11^
I-product	*Enterococcus faecium*	NCIMB 10415	2^*^10^11^
J-product	*Lactobacillus plantarum*	DSM 12837	1^*^10^9^
	*Pediococcus acidilactici*	DSM 16243	
	*Enterococcus faecium*	DSM 7134	

The classification of strains into categories is overseen by three main international organizations. Based on their standards, probiotic strains found in products are provided with unique identifiers. The CECT (Spanish Type Culture Collection) is a Spanish strain collection accredited with ISO 9001 standards (24). The NCIMB (National Collection of Industrial, Food and Marine Bacteria) is a privately-owned strain collection located in the United Kingdom (25). The DSM (Deutsche Sammlung von Mikroorganismen und Zellkulturen GmbH) is a German strain collection (26).

### 2.2 Preparation of antibiotics solution

Strain solutions were prepared from the active ingredients in accordance with the recommendations of the Clinical Laboratory Standards Institute (CLSI), utilizing materials from Merck KGaA, Darmstadt, Germany (27). For the experiments, strain solutions were prepared at a concentration of 1,024 μg/mL, with adjustments made for the purity specified by the manufacturer of each active ingredient. The experiments were conducted within a 2-fold dilution range, spanning from 128 to 0.125 μg/mL.

### 2.3 Determining the minimum inhibitory concentration

The phenotypic expression of resistance was examined by determining the minimum inhibitory concentration (MIC) values of each bacterial strain, which was conducted using the CLSI methodology (27). The breakpoints were determined based on the guidelines of both the CLSI and the European Committee on Antimicrobial Susceptibility Testing (EUCAST) (28). The selection of antimicrobial agents was made based on international literature, aiming to cover the most frequently used antimicrobial groups.

The bacterial strains were stored at −80°C and inoculated into 3 ml of Mueller-Hinton broth (MHB) the day before the experiment, followed by incubation at 37°C for 18–24 h. The experiments were conducted using a 96-well microtiter plate (VWR International, LLC., Debrecen, Hungary). The first column of the working plates, filled with 90 μl of MHB, received a 4-fold dilution of the strain solutions, establishing an initial concentration of 128 μg/mL, from which a 2-fold dilution series was prepared, except in the last two columns of 96-well microtiter plate. The penultimate column served as a positive control (containing only bacteria), while the final column served as a negative control (containing only the drug). Bacterial suspension adjusted to 0.5 McFarland turbidity was inoculated onto the working plates up to the positive control column using a nephelometer (CheBio fejleszto Kft., Budapest, Hungary) (27). Evaluation took place after 24 h of incubation at 37°C using the SWIN automatic MIC reader (CheBio fejleszto Kft., Budapest, Hungary) and the VIZION system (CheBio fejleszto Kft., Budapest, Hungary). The reference isolate used was *Escherichia coli* (ATCC 25922).

### 2.4 Next-generation sequencing and bioinformatic analysis

Probiotic samples were prepared by using 0.1 g of probiotic powder dissolved in 1 ml PBS (1:10 dilution). Nucleic acid was extracted from the mixture using the Quick-DNA Fecal/Soil Microbe Miniprep Kit (Zymo Research, CA, USA) according to the manufacturer's instruction. Samples were disrupted by using a TissueLyzer LT Bead Mill (Qiagen, Germany). The concentration of purified DNA was measured with Qubit 2.0 equipment using the Qubit dsDNA BR Assay Kit (Thermo Scientific, Waltham, MA, USA).

Nucleotide sequences were determined by next-generation sequencing on an Illumina^®^ NextSeq 500 sequencer (Illumina, San Diego, CA, USA) following the reference guide provided by Illumina. Illumina^®^ Nextera XT DNA Library Preparation Kit (Illumina, San Diego, CA, USA) and Nextera XT Index Kit v2 Set A (Illumina, San Diego, CA, USA) were used to prepare Illumina specific libraries. DNA samples were diluted to 0.2 ng/μL in nuclease-free water (Promega, Madison, WI, USA) in a final volume of 2.5 μL. Reaction components were used at a reduced volume. For the tagmentation reaction, 5 μL Tagment DNA buffer with 2.5 μL AmpliconTagment Mix were used. During tagmentation, the samples were incubated at 55°C for 6 min, using the GeneAmp PCR System 9700 (Applied Biosystems/Thermo Fisher Scientific, Foster City, CA, USA). The samples were then allowed to cool to 10°C before the addition of 2.5 μL of the Neutralize Tagment buffer. Neutralization was performed for 5 min at room temperature. A total of 7.5 μL of the Nextera PCR Master Mix and 2.5 μL each of the i5 and i7 index primers were added to the tagmented DNA samples. The index primers were attached to library DNA via 12 PCR cycles (each cycle consisted of the following steps: 95°C for 10 s, 55°C for 30 s, followed by 72°C for 30 s). Following the PCR cycles, the samples were held at 72°C for 5 min and then at 10°C. Libraries were purified using Gel/PCR DNA Fragments Extraction Kit (Geneaid Biotech Ltd., Taipei, Taiwan). The concentration of the purified libraries was measured with Qubit 2.0 equipment using Qubit dsDNA HS Assay Kit (Thermo Fischer Scientific, Waltham, MA, USA). Library DNAs were pooled and denatured. The denatured library pool was loaded onto a NextSeq 500/550 Mid Output flowcell at a final concentration of 1.5 pM. Sequencing was performed using an Illumina^®^ NextSeq 500 sequencer (Illumina, San Diego, CA, USA).

During the bioinformatic data processing, quality control of the raw sequences was performed using FastQC v0.11.9 (29), followed by the removal of low-quality sections using TrimGalore v0.6.6 (30). The reads were assembled into longer sequences (contigs) using MEGAHIT v1.2.9 (31). From these contigs, all possible open reading frames (ORFs) were determined using Prodigal v2.6.3 (32). Protein sequences were derived from these ORFs based on their nucleotide sequences. Subsequently, the protein sequences were compared to antimicrobial resistance gene (ARG) sequences in The Comprehensive Antibiotic Resistance Database (CARD) using the Resistance Gene Identifier (RGI) v5.1.0 software (33). Only results reaching the specified threshold (95%) in the CARD database were retained.

The potential mobility of identified resistance genes was assessed using the MobileElementFinder v1.0.3 program (34) which predicted genes occurring as mobile genetic elements (MGEs) on contigs. Only those ARGs found within the longest composite transposon distance specific to the species in the database within the contig were considered as potentially mobile. Additionally, plasmid encoding was examined using the PlasFlow v1.1 software, and the presence of phage genomes on contigs was determined using the VirSorter v2.2.2 (35) software.

## 3 Results

### 3.1 *Enterococcus faecium* strains

Table 2 summarizes the results of susceptibility testing of *Enterococcus faecium* strains isolated from the examined products. All the products contained the same strain (NCIMB10415) except for the J-product (DSM7134). Similar MIC values were observed for all strains. All the tested strains were sensitive to penicillin, amoxicillin, amoxicillin-clavulanate, oxytetracycline, doxycycline, clindamycin, tylosin, and vancomycin. For gentamicin (100%; MIC >32 μg/mL) and potentiated sulfonamides (sulfamethoxazole and trimethoprim in a 19:1 ratio; 100%; MIC 16 μg/mL), all strains were resistant. Regarding gatifloxacin, a fourth-generation fluoroquinolone, six strains were susceptible, while three were resistant (66.7%; MIC >2 μg/mL).

**Table 2:** Minimum inhibitory concentration (MIC) values of *Enterococcus faecium* strains isolated from the products for tested antibiotics.

**Products**	**Animal species**	**Species**	**PEN**	**AMX**	**AMC**	**GEN**	**OTC**	**DOX**	**CLI**	**PSA**	**GAT**	**FLO**	**TIL**	**VAN**
		**Breakpoint** ^*^	**MIC (**μ**g/mL)**											
			≥**16**	≥**8**	≥**8**	≥**32**	≥**8**	≥**0.5**	≥**32**	≥**16**	≥**2**	≥**8**	≥**128**	≥**4**
B-product	Dogs, cats	*Enterococcus faecium*	4	1	1	>32	0.25	<0.125	4	>128	0.5	8	2	1
C-product	Dogs, cats		8	1	1	>32	0.25	<0.125	4	>128	0.5	8	2	1
D-product	Cats		4	1	2	32	0.25	<0.125	4	>128	1	8	1	1
E-product	Dogs		8	1	2	32	0.25	<0.125	4	>128	0.5	8	1	2
F-product	Dogs, cats		4	1	1	>32	0.25	<0.125	4	>128	0.5	8	2	2
G-product	Dogs, cats		8	1	1	>32	0.25	<0.125	4	>128	0.5	8	2	1
H-product	Dogs		8	1	1	>32	0.25	<0.125	8	>128	2	8	1	1
I-product	Dogs, cats		8	1	1	>32	0.25	<0.125	4	>128	2	8	2	2
J-product	Dogs		8	1	1	32	0.25	<0.125	8	>128	2	8	1	1

PEN, penicillin; AMX, amoxicillin; AMC, amoxicillin-clavulanic acid; GEN, gentamicin; OTC, oxytetracycline; DOX, doxycycline; CLI, clindamycin; PSA, potentiated sulphonamide; GAT, gatifloxacin; FLO, florfenicol; TIL, tylosin; VAN, vancomycin.

^*^CLSI and EUCAST.

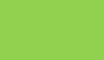
sensitive 
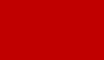
resistant.

### 3.2 *Lactobacillus* and *Pediococcus* strains

Table 3 summarizes the antibiotic susceptibility profiles of *Lactobacillus* and *Pediococcus* species. Among the *Lactobacillus* strains, one *Lactobacillus plantarum* strain was isolated, which showed sensitivity to penicillin, amoxicillin-clavulanate, gentamicin, oxytetracycline, doxycycline, clindamycin, tylosin, and vancomycin. It was resistant to amoxicillin (>1 μg/mL MIC), the combination of sulphonamides (>16 μg/mL MIC), and gatifloxacin (>2 μg/mL MIC). From the *Pediococcus* species, two *Pediococcus pentasaceus* and one *Pediococcus acidilactici* strains were isolated, all of which were susceptible only to clindamycin (< 8 μg/mL MIC), showing resistance to all other tested antibiotics.

**Table 3:** Minimum inhibitory concentration (MIC) values of *Lactobacillus* and *Pediococcus* strains isolated from the products for tested antibiotics.

**Products**	**Animal species**	**Species**	**PEN**	**AMX**	**AMC**	**GEN**	**OTC**	**DOX**	**CLI**	**PSA**	**GAT**	**FLO**	**TIL**	**VAN**
			**MIC (**μ**g/mL)**											
		**Breakpoint** ^*^	≥**8**	≥**8**	≥**8**	≥**512**	≥**32**	≥**32**	≥**8**	≥**32**	≥**2**	**-**	≥**32**	≥**16**
A-product	Dogs	*Lactobacillus plantarum*	1	16	0.25	8	4	2	< 0.125	>128	2	2	2	1
**Products**	**Animal species**	**Breakpoint** ^*^	**≥0.125**	**≥0.125**	**≥0.125**	**≥4**	**≥4**	**≥4**	**≥0.5**	**≥2**	**≥1**	**≥8**	**-**	**≥4**
D-product	Cats	*Pediococcus pentasaccus*	0.5	16	4	>128	32	16	< 0.125	>128	8	8	2	>128
E-product	Dogs	*Pediococcus pentasaccus*	1	16	4	32	32	32	< 0.125	>128	8	8	4	>128
G-product	Dogs, cats	*Pediococcus acidilactici*	0.25	8	2	>128	32	8	< 0.125	>128	4	8	2	>128

PEN, penicillin; AMX, amoxicillin; AMC, amoxicillin-clavulanic acid; GEN, gentamicin; OTC, oxytetracycline; DOX, doxycycline; CLI, clindamycin; PSA, potential sulphonamide; GAT, gatifloxacin; FLO, florfenicol; TIL, tylosin; VAN, vankomicin.

^*^CLSI and EUCAST.

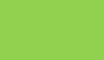
sensitive 
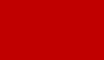
resistant.

### 3.3 Results of metagenomic analysis

Table 4 summarizes the results obtained from next-generation sequencing, including the isolated resistance genes from the products, their taxonomic origin, indicating the ARG family for each gene, the resistance mechanism determined by the respective gene, and the antibiotic groups against which resistance is conferred.

**Table 4:** The antimicrobial resistance gene (ARG) set of each product, their mechanism of action, resistance to the antibiotics group.

**Gene**	**Taxon origin**	**ARG family**	**Mechanism**	**I**	**II**	**III**	**IV**	**V**	**VI**	**VII**	**VIII**	**IX**	**X**	**XI**	**B^a^**	**C^a^**	**D^a, b, c^**	**E^a, b, c^**	**F^a, d, e^**	**G^a, d, e^**	**H^a^**	**I^a^**	**J^b, c, f^**
*AAC(6′)-Ii*	*Enterococcus* spp.	Acetyltransferase	Enzymatic inactivation		x										x	x	x	x	x	x	x	x	x
*aad(6)*	*Lactococcus* spp.	Nucleotidyltransferase	Enzymatic inactivation		x										x							x	
*APH(3′)-Ia*	*Klebsiella* spp.	Phosphotransferase	Enzymatic inactivation		x										x							x^*^	
*APH(3′')-Ib*	*Klebsiella* spp.	Phosphotransferase	Enzymatic inactivation		x										x							x	
*APH(3′)-IIIa*	*Lactococcus* spp.	Phosphotransferase	Enzymatic inactivation		x										x							x	
*APH(6)-Id*	*Pseudomonas* spp.	Phosphotransferase	Enzymatic inactivation		x										x							x	
*catA8*	*Lactobacillus* spp.	Acetyltrasnferase	Enzymatic inactivation				x															x	
*CRP*	*Hafnia* spp.	RND-type	Efflux pump	x				x			x				x								
*eatAv*	*Enterococcus* spp.	ABC-F	Target protection						x	x					x	x	x	x	x	x	x	x	x
*efmA*	*Enterococcus* spp.	MFS-type	Efflux pump					x			x						x	x					x
*ermB*	*Enterococcus* spp.	Methyltransferase	Target modification					x	x						x							x	
*EF-Tu*	*Hafnia* spp.	ABC-F	Target protection												x								
*msrC*	*Enterococcus* spp.	ABC-F	Target protection					x							x	x	x	x	x	x	x	x	x
*rsmA*	*Hafnia* spp.	RND-type	Efflux pump				x				x	x			x								
*SAT-4*	*Lactococcus* spp.	Acetyltransferase	Enzymatic inactivation			x								x	x							x	
*tetC*	*Enterococcus* spp.	MFS-type	Efflux pump			x																x	
*tetD*	*Acinetobacter* spp.	MFS-type	Efflux pump			x									x								
*tetM*	*Enterococcus* spp.	Mosaic, ribosome	Target protection			x																x	
*tetS*	*Streptococcus* spp.	Mosaic, ribosome	Target protection			x									x^*^							x	

In addition to resistance genes, their taxonomic origin, and the mechanism of action and gene family, it is also indicated which antibiotic class the expression of the given gene confers resistance to, as well as in which products it was found. Cases where we assume the phenotypic expression of the gene based on minimum inhibitory concentration (MIC) values indicating phenotypic resistance are highlighted in purple. Cases where the resistance gene was identified on a plasmid are highlighted in green, and an asterisk is used to mark genes that were mobile genetic elements (MGEs).

I. penicillins, II. aminoglycosides, III. tetracyclines, IV. phenicols, V. macrolides, VI. llinkosamides, VII. pleuromutilins, VIII. fluoroquinolones, IX. diaminopyrimidines, X. rifamicins, and XI. nucleocides.

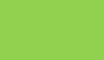
genes on plasmids, 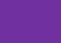
phenotypic expression of a given ARG in the light of MIC values.

^*^MGE, mobile genetic element.

^a^E. faecium (NCIMB10415).

^b^L. plantarum (DSM12837).

^c^P. acidilactici (DSM1283U).

^d^L. plantarum (DSM12836).

^e^P. acidilactici (DSM1283U).

^f^E. faecium (DSM7134).

A total of 19 types of ARGs were identified, out of which 11 were found on plasmids, and no genes were identified on phages. Of particular concern is that in two cases, a plasmid-contained gene also acted as a mobile genetic element (MGE). One of these was the *APH(3*′*)-Ia* gene, found in the I-product. The activation of this resistance gene through enzymatic means (phosphotransferase) can inactivate aminoglycoside antibiotics (36). The other gene was the *tetS* gene, found in the A-product. This gene is capable of reducing sensitivity to tetracycline antibiotics through target protection (ribosomal mosaic), thereby preventing the binding of the antibiotic (37).

During the determination of MIC values, a correlation can be observed between resistant values and the identified ARGs in the *Enterococcus faecium* strains of various products. The expression of the *AAC(6*′*)-Ii* gene enzymatically (acetyl-transferase) leads to resistance to aminoglycoside antibiotics. This gene was found in all tested products and could be one of the genes responsible for resistance (≥32 μg/mL) observed in all strains. It's typically chromosomally located, but in one case (F-product), it was found on a plasmid. The *eatAv* gene confers resistance to lincosamides and pleuromutilins through target protection (ABC-F type). This gene was present in all *Enterococcus faecium* strains from the tested products. The *efmA* gene determines an MFS-type efflux pump for macrolides and fluoroquinolones, potentially contributing to resistance to gatifloxacin (2 μg/mL MIC) observed in one case (J-product). The *rsmA* gene determines an RND-type multidrug efflux pump, which can confer resistance to phenicols, fluoroquinolones, and diaminopyrimidines. This gene was also found in all *Enterococcus faecium* strains and may explain the observed resistance (>128 μg/mL MIC) to potentiated sulfonamides, gatifloxacin (2 μg/mL MIC) in the H-product, I-product, and J-product cases, as well as potential florfenicol resistance (8 μg/mL MIC).

All phenotypic resistances were successfully attributed to identified ARGs. Of particular concern is the high proportion of genes found on plasmids (57.9%), with two instances where these were also MGEs. Therefore, based on our findings, it would be necessary to conduct such examinations, similar to those required for food-producing animals, before introducing probiotic preparations intended for companion animals into circulation. In product A, containing strains of *Lactobacillus plantarum* (NCIMB41638), *Lactobacillus fermentum* (NCIMB41636), and *Lactobacillus rhamnosus* (NCIMB41640), no ARGs were found. Therefore, the phenotypic resistance observed in this case cannot be genetically supported by detected genes.

The B-product contained *Enterococcus faecium* (NCIMB10415), from which we identified 15 ARGs, out of which seven were located on plasmids and one (*tetS*) gene was on an MGE. Four genes were specifically derived from *Enterococcus* species, while the rest originated from *Lactococcus, Klebsiella, Pseudomonas, Hafnia, Acinetobacter*, and *Streptococcus* species. Similarly, in product C and product H containing *Enterococcus faecium* (NCIMB10415), we identified 3 ARGs, all of which were chromosomal genes originating from *Enterococcus* spp. The D-product, E-product, and J-product containing *Lactobacillus plantarum* (DSM12837), *Pediococcus acidilactici* (DSM16243), and *Enterococcus faecium* (NCIMB10415) species, respectively, all showed the presence of the same four ARGs. These genes were chromosomally located, and all originated from *Enterococcus* spp.

The F-product contained strains of *Pediococcus pentasaceus* (DSM1283U), *Lactobacillus brevis* (DSM12835), *Lactobacillus bucherii* (DSM12856), *Lactobacillus plantarum* (DSM12836), *Lactobacillus rhamnosus* (NCIMB30121), *Lactobacillus acidophilus* (CECTU529), and *Enterococcus faecium* (NCIMB10415), with *Pediococcus* and *Lactobacillus* species present in deactivated form in the product. Four resistance genes matched those found in the E-product. However, in the case of the F-product, the *AAC(6*′*)-Ii* gene was located on a plasmid, while the rest were chromosomal. All identified genes were attributed to *Enterococcus* spp. The composition of the G-product was identical to that of the F-product, but only two chromosomally located ARGs originating from *Enterococcus* spp. could be identified in the tablets.

The I-product, which contained the *Enterococcus faecium* (NCIMB10415) strain, contained the most identified ARGs, totaling 14. Of these, eight were found on plasmids, and among these, the *APH(3*′*)-Ia* gene was also an MGE. Five of the genes were originally of *Enterococcus* spp. origin, while the rest may have been acquired from other species through horizontal gene transfer, including *Staphylococcus, Klebsiella, Lactobacillales, Enterobacteriaceae, Enterobacterales*, and *Streptococcus* species.

## 4 Discussion

The *in vitro* antibiotic susceptibility of probiotic strains isolated from a total of 10 commercially available probiotic products for companion animals (dogs, cats) was investigated, and 19 different ARGs were identified during the exploration of the products through next-generation sequencing.

Gentamicin resistance was observed in all strains of *Enterococcus faecium* examined in our study, with MIC values >32 μg/mL. In contrast, in the study conducted by Takeuchi et al., this value exceeded 500 μg/mL in 22% of the isolated *Enterococcus faecium* strains (38). Extremely high values (9,000 μg/mL MIC) were detected in a strain isolated from wastewater by Xu et al. during antimicrobial susceptibility testing against sulphonamide (sulfamethoxazole-trimethoprim) (39). Significantly lower values (16 μg/mL MIC) were identified in our research. Similarly, comparable results for both gentamicin and sulphonamide susceptibility were obtained in isolates from poultry (32 μg/mL MIC, 16 μg/mL MIC) by Maasjost et al (40). Resistance to gatifloxacin was also observed in three strains, albeit at relatively low levels (>2 μg/mL MIC). This finding is supported by several previous studies. Wenzler et al. worked with clinical isolates, where the median value (2 μg/mL MIC) matched those described in our study (41).

Nine of the preparations we examined contained *Enterococcus faecium*, in which all 19 types of ARG were present. Xia et al. identified 18 ARGs in *Enterococcus faecium* strains, none of which were MGE (42), in contrast, we described the likelihood of mobility for two genes [*APH(3*′*)-Ia, tetS*]. Several of the genes we identified confer resistance to aminoglycosides through different enzymatic pathways. The *AAC(6*′*)-Ii* gene is an aminoglycoside transferase found on the chromosome, first described in *Enterococcus faecium* (43). The *AAD(6)* gene, found on a plasmid, encodes aminoglycoside nucleotide transferase (44). The *APH(3*′*)* gene family members confer aminoglycoside resistance through enzymatic inactivation. The *APH(3*′*)-Ia* gene is found on a transposon in *Escherichia coli* and *Salmonella* species (36). The *APH(3*′*)-IIIa* gene is plasmid-encoded, found in *Staphylococcus aureus* and *Enterococcus* species (45). The *APH(3*′*)-Ib* gene was identified in *Escherichia coli*, also found on a plasmid (46). The *APH(6)-Id* operates through a similar mechanism. This gene can be found on a plasmid, transposon, or chromosome (47).

The *catA8* gene encodes a chloramphenicol acetyltransferase enzyme. Through enzymatic inactivation, it neutralizes phenicols, primarily chloramphenicol (48). Metagenomic analysis of the *catA8* gene identified by us supported its origin in *Lactobacillus* and its presence on a plasmid. The *eatAv* gene was first identified in *Enterococcus faecium* bacteria. Its mechanism involves target protection, conferring resistance to pleuromutilins and lincosamides (49). The expression of *ermB* is induced by the erythromycin agent. As a result, resistance is developed against macrolides and lincosamides through modification of the target (50).

The *msrC* gene, typically found in *Enterococcus faecium* strains, is a chromosomally encoded gene that confers resistance primarily to macrolides, specifically erythromycin. Through target protection, it prevents the action of antibiotics (51). We observed phenotypic expression of this gene in most cases based on MIC values, and in one preparation, we identified it on a plasmid. Urshev et al. detected the *msrC* gene in an *Enterococcus faecium* strain (52). Thumu et al. first identified the *msrC* gene in *Pediococcus pentasaceus* species, but they also detected the presence of the *ermB* gene (53). In our investigations, we identified both genes in several preparations, but based on metagenomic analysis, we found that these genes were of *Enterococcus* spp. origin. However, phenotypically, we observed the expression of resistance as defined by them. The *rsmA* gene encodes a small RNA-binding protein that plays a post-transcriptional regulatory role in shaping the virulence genes of *Pseudomonas aeruginosa*. It confers resistance to diaminopyrimidines, phenicols, and fluoroquinolones through an efflux pump mechanism (54). The *SAT-4* gene, derived from *Campylobacter coli* bacteria, is a plasmid-mediated streptothricin acetyltransferase (55).

The *tetC* gene is responsible for conferring resistance to tetracycline antibiotics, primarily in Gram-negative bacteria, as it regulates the expression of an efflux pump gene, usually found on plasmids. The *tetD* gene is similar but is exclusively present in Gram-negative species. Resistance is developed through target protection mechanisms (56). The *tetM* and *tetS* genes are responsible for ribosome protection proteins and are located on mobile genetic elements, found in both Gram-negative and Gram-positive bacteria. Similar to Pan et al. (57) we also identified the *tetM* gene on a plasmid, but during this study, it was identified as originating from Enterococcus. The *tetS* gene we detected proved to be of *Streptococcus* spp. origin, found on a plasmid, and was mobile in the case of B-product. Nawaz et al. identified these genes during their study of *Lactobacillus plantarum*, successfully transferring the genes into *Enterococcus* isolates under experimental conditions (13). In our investigations, the *ermB* gene identified in preparations containing *Lactobacillus plantarum* always proved to be of *Enterococcus* spp. origin.

Based on the results, it is recommended in practice to consider introducing stricter regulations during the production and marketing authorization of probiotics, ensuring the reduction of the ARG pool in products to a minimal level, with particular attention to the exclusion of genes that may have significant public health importance. Preliminary resistance profile assessments during the selection of probiotic strains reduce the likelihood of spreading resistances that can be easily transferred, creating a bridge between animal and public health, and posing a significant risk through their use. In every case of probiotics, it would be necessary to compile a panel of antibiotic active substances based on phenotypic test results, upon which genetic background exploration would be conducted using next-generation sequencing, with particular attention to the carriage of ARGs on plasmids or phages, as well as their encoding as MGEs. The exclusion of these is essential for minimizing the chances of horizontal gene transfer. The limitations of our studies include that they were conducted with only a few products involved. In the future, it is definitely worthwhile to involve a broader range of products in the studies and to conduct more parallel investigations to enable statistical analyses.

## 5 Conclusion

We can conclude that among the probiotic strains we studied, *Enterococcus faecium* bacteria carry the most resistance genes, in accordance with the existing literature. Among *Lactobacillus* and *Pediococcus* species, we identified resistance genes of *Enterococcus* strains in most cases, also consistent with literature. We detected a *Lactobacillus plantarum*-derived gene (*efmA*) responsible for resistance, which has not been previously described. The majority of identified ARGs (57.9%) were found on plasmids; in two preparations, B-product (*tetS* gene) and I-product [*APH(3*′*)-Ia* gene], we identified plasmid-borne ARGs, also serving as MGE genes. The literature on this topic is relatively scarce, particularly regarding studies exploring the ARG repertoire in probiotic preparations intended for companion animals. Our results underscore the importance of conducting such studies, and it may be worth considering legally mandating such investigations as a condition for distribution, similar to legislation implemented for products intended for livestock.

## Data Availability

The datasets presented in this study can be found in online repositories. The names of the repository/repositories and accession number(s) can be found in the article/Supplementary material.
