# Corrigendum: Investigating antimicrobial resistance genes in probiotic products for companion animals

**DOI:** 10.3389/fvets.2024.1531511

**Published:** 2024-12-17

**Authors:** Adam Kerek, Emese Szabó, Ábel Szabó, Márton Papp, Krisztián Bányai, Gábor Kardos, Eszter Kaszab, Krisztina Bali, Ákos Jerzsele

**Affiliations:** ^1^Department of Pharmacology and Toxicology, University of Veterinary Medicine, Budapest, Hungary; ^2^National Laboratory of Infectious Animal Diseases, Antimicrobial Resistance, Veterinary Public Health and Food Chain Safety, University of Veterinary Medicine, Budapest, Hungary; ^3^Centre for Bioinformatics, University of Veterinary Medicine, Budapest, Hungary; ^4^Veterinary Medical Research Institute, Budapest, Hungary; ^5^One Health Institute, Faculty of Health Sciences, University of Debrecen, Debrecen, Hungary; ^6^National Public Health Center, Budapest, Hungary; ^7^Department of Metagenomics, University of Debrecen, Debrecen, Hungary; ^8^Department of Microbiology and Infectious Diseases, University of Veterinary Medicine, Budapest, Hungary

**Keywords:** probiotics, ARG, NGS, companion animals, antimicrobial resistance

In the published article, there was an error in Table 2 as published. An incorrect letter was used in the first column of the table. In the fourth row of the first column, “A-product” was incorrectly indicated. The correct product is “B-product.” The corrected Table 2 and its caption appear below.

**Table 2:** Minimum inhibitory concentration (MIC) values of *Enterococcus faecium* strains isolated from the products for tested antibiotics.

**Products**	**Animal species**	**Species**	**PEN**	**AMX**	**AMC**	**GEN**	**OTC**	**DOX**	**CLI**	**PSA**	**GAT**	**FLO**	**TIL**	**VAN**
		**Breakpoint** ^*^	**MIC (**μ**g/mL)**											
			≥**16**	≥**8**	≥**8**	≥**32**	≥**8**	≥**0.5**	≥**32**	≥**16**	≥**2**	≥**8**	≥**128**	≥**4**
B-product	Dogs, cats	*Enterococcus faecium*	4	1	1	>32	0.25	< 0.125	4	>128	0.5	8	2	1
C-product	Dogs, cats		8	1	1	>32	0.25	< 0.125	4	>128	0.5	8	2	1
D-product	Cats		4	1	2	32	0.25	< 0.125	4	>128	1	8	1	1
E-product	Dogs		8	1	2	32	0.25	< 0.125	4	>128	0.5	8	1	2
F-product	Dogs, cats		4	1	1	>32	0.25	< 0.125	4	>128	0.5	8	2	2
G-product	Dogs, cats		8	1	1	>32	0.25	< 0.125	4	>128	0.5	8	2	1
H-product	Dogs		8	1	1	>32	0.25	< 0.125	8	>128	2	8	1	1
I-product	Dogs, cats		8	1	1	>32	0.25	< 0.125	4	>128	2	8	2	2
J-product	Dogs		8	1	1	32	0.25	<0.125	8	>128	2	8	1	1

PEN, penicillin; AMX, amoxicillin; AMC, amoxicillin-clavulanic acid; GEN, gentamicin; OTC, oxytetracycline; DOX, doxycycline; CLI, clindamycin; PSA, potentiated sulphonamide; GAT, gatifloxacin; FLO, florfenicol; TIL, tylosin; VAN, vancomycin.

* CLSI and EUCAST.

▭ sensitive ▭ resistant.

The authors apologize for this error and state that this does not change the scientific conclusions of the article in any way. The original article has been updated.

